# Correlation between antiphospholipid antibodies and renal involvement in children with Henoch-Schönlein purpura: A cross-sectional study

**DOI:** 10.22088/cjim.15.2.287

**Published:** 2024

**Authors:** Mehrnoush Hassas Yeganeh, Reza Sinaei, Aye Yaraghi, Khosro Rahmani, Vadood Javadi Parvaneh, Reza Shiari, Hamid Hosseinzadeh

**Affiliations:** 1 Pediatric Pathology Research Center, Research Institute for Children Health, Shahid Beheshti University of Medical Sciences, Tehran, Iran; 2 Department of Pediatrics, School of Medicine, Kerman University of Medical Sciences, Kerman, Iran; Clinical Research Development Unit, Afzalipour Hospital, Kerman University of Medical Sciences, Kerman, Iran; 3 Mofid Children Hospital, Shahid Beheshti University of Medical Sciences, Tehran, Iran; 4 Rowan University, Glassboro, NJ, USA

**Keywords:** Child, IgA vasculitis, Henoch-Schönlein purpura, Antiphospholipid antibody.

## Abstract

**Background:**

Renal involvement is the most damaging long-term complication of Immunoglobulin-A (IgA) vasculitis. In the lack of a definite predictive biomarker for renal involvement, antiphospholipid antibodies (aPL) have been proposed in recent years.

**Methods:**

In this prospective cohort of 48 pediatric patients who were admitted with IgA vasculitis from September 2015 to June 2017, two serum samples were taken 12 weeks apart to detect Anti-Phospholipid antibodies. All patients were followed-up for renal involvement for six months.

**Results:**

Renal involvement occurred in 14 out of 48 patients with IgA vasculitis (29.16%). APLs were positive in nine out of 14 patients with IgA vasculitis and renal involvement (64.28%), in contrast to only six out of 34 patients with IgA vasculitis without renal involvement (17.64%). The presence of aPL antibodies was statistically associated with renal involvement (P=0.002). Although, the relationship between both sex (P=0.025) and age (P=0.046) with aPL positivity was statistically significant, performing a modified logistic regression test, the odds ratio was significant between the groups with and without renal involvement only in term of age and aPL positivity).

**Conclusion:**

The presence of aPL antibodies was statistically associated with renal involvement. We found a significant relationship between the age and aPL positivity. Hence, we need multicenter, more extensive cohort studies to reach a better and more accurate conclusion on the relationship between serum aPLs and renal involvement in IgA vasculitis patients.

IgA vasculitis is a hypersensitivity vasculitis characterized by the deposition of IgA-containing immune complexes within the blood vessels throughout the body. The main clinical features include non-thrombocytic purpura (with lower limb dominance), abdominal pain, arthritis/ arthralgia, and renal involvement (1). 

Renal involvement is the most threatening long-term complication of IgA vasculitis, occurring in 20-80% of affected children, so that 80% of them present with isolated microscopic or macroscopic hematuria and/or proteinuria, whereas only 20% have acute nephritis or nephrotic syndrome at presentation (2). Whereas, other organs involvement in Henoch-Schönlein purpura (HSP) are generally benign and self-limited, the renal involvement seems more serious and usually develops within four to eight weeks after the initial symptoms of HSP (3). 

Most patients with renal involvement have a favorable prognosis. However, around 20% of these patients develop end-stage renal disease (ESRD) (2 ,4). IgA vasculitis nephritis is the only disease manifestation that may be associated with long-lasting morbidity; hence, the long-term prognosis of IgA vasculitis is mainly determined by the progression of renal involvement (4, 5).

Some factors have been reported to be associated with renal involvement in IgA vasculitis, such as age more than 10-years at onset, male gender, digestive tract symptoms, persistency (> one month) or recurrence of purpura, white blood cell (WBC) count>15X109/L, platelet count >500X109/L, lymphopenia, upper respiratory tract infection, especially streptococcal infection with elevated Anti Streptolysin-O (ASO) titer, and decreased C3 level (4, 6).

Onset at an older age (>6 years), longer interval between IgA vasculitis symptom onset and IgA vasculitis diagnosis (>8 days), presence of angioedema, recurrence of purpura, and Central Nervous System (CNS) involvement are factors associated with increased risk of severe kidney disease (1). However, no biomarker has been proven as a risk factor for renal involvement and its severity yet (7). 

Association between IgA vasculitis and aPL antibodies has been first reported in 2000 (8). Since then, the presence of aPL antibodies in patients with IgA vasculitis has been reported to be associated with disease activity (9, 10), and central nervous system involvement (11). However, the association between aPL antibodies and renal involvement and prognosis in IgA vasculitis remains unrevealed.

In the present study, to confirm the correlation between renal involvement in IgA vasculitis and aLP antibodies, we report the 6-month follow-up of 48 patients with IgA vasculitis. 

## Methods

**Study design and Clinical investigation: **In this prospective cohort, 48 pediatric patients including 31 boys and 17 girl with a mean age of 6.29 ± 1.35 (5-9) years were enrolled to the study.

According to 2008 criteria of European League Against Rheumatology/ Pediatric Rheumatology European Society (EULAR/ PRES) for diagnosis of IgA vasculitis (palpable purpura as the mandatory criterion, in the presence of at least one of the following four features of diffuse abdominal pain, any biopsy showing predominant IgA deposition, arthritis or arthralgia, and renal involvement as the presence of any hematuria and/or proteinuria) (12), 48 pediatric patients who were diagnosed and admitted with IgA vasculitis at Mofid Children Hospital (SBMU, Tehran, Iran) from September 2015 to June 2017 were enrolled to the study. 

We considered the following criteria for diagnosing renal involvement in IgA vasculitis: 1) both microscopic (apparent only upon urinalysis) and gross (visible to the naked eye) hematuria in urine analysis, 2) proteinuria in urine analysis; and 3) both proteinuria and hematuria (12). We followed all patients for six months since children with no urinary pathology during the first six months after the onset of IgA vasculitis will not develop renal impairment during the long term follow-up (13). All patients were admitted and diagnosed by pediatric rheumatology subspecialists, and their information was collected and followed by an expert rheumatologist. Skin biopsy of the site of vasculitis in the leg was performed in suspected cases to rule out of other differential diagnosis. Skin biopsies with neutrophilic infiltration in the small vessels wall, perivascular neutrophilic infiltration in the dermis, endothelial swelling, perivascular edema with Red Blood Cells (RBC) extravasation, and leukocytoclastic vasculitis and nuclear derbies were compatible with diagnosis.

**Inclusion and Exclusion Criteria:**

**Inclusion criteria:** All pediatric patients under 18-years-old who presented with typical characteristics of non-thrombocytopenic palpable purpura over the lower extremities compatible with the diagnosis of HSP (12) and admitted in Mofid Children Hospital, Tehran, Iran between September 2015 and June 2017 were enrolled to the study. 

**Exclusion Criteria: **All patients who did not fulfill the 2008 EULAR/ PRES criteria for diagnosis of IgA vasculitis and patients more than 18-years-old did not enroll in the study. In addition, patients who were diagnosed with other diseases including systemic lupus or other vasculitis, were excluded from the study.

**Detection of antiphospholipid antibodies:** We obtained two serum samples from all patients with IgA vasculitis to detect aPL antibodies; the first sample on the time of patient admission, and the second one, 12 weeks later, to make sure that the aPL antibodies are still elevated. All samples were preserved at -80 ˚C, no more than four weeks before the detection of antiphospholipid antibodies. Laboratory assessment of antiphospholipid antibodies was based on the latest update of the classification criteria for definite antiphospholipid antibody syndrome (14). Lupus anticoagulant was detected with the Kaolin conglutination time method, whereas anti-cardiolipin antibodies (M & G) and anti-b2 glycoprotein-I antibodies (M & G) were detected with AESKU, Germany kits by enzyme-linked immunosorbent assays (ELISA). The test results were interpreted as the normal range of these antibodies less than 20 units, and more than 20 units as the positive cases. Detection of positive titers of even one of these antiphospholipid antibodies was recognized as a positive result.

**Statistical methods: **Statistical analyses were performed using SPSS Version 22.0 (IBM, New York, NY, USA). The t-test was performed for quantitative variables and chi-square test, Pearson correlation and Fisher’s exact tests were used for qualitative variables to compare the rate of positive aPL in patients with IgA vasculitis, with and without renal involvement. A p-value of less than 0.05 was regarded as statistically significant. Both Crude and adjusted odds ratios for estimating risk and controlling for confounding bias were evaluated in this study.

**Ethics Approval and Consent to Participate: **The Ethics Committee of Shahid Beheshti Medical University (SBMU) approved this study, in accordance with ethical standards of the local committee of the SBMU’s ethics board and the Declaration of Helsinki. The ethical approval number is IR.SBMU.RETECH.REC.1397.595. We obtained informed consent from the parents of all patients.

## Results

We included 48 patients with a confirmed diagnosis of IgA vasculitis based on clinical criteria. 31 out of 48 patients were boys with a boy to girl ratio of 1.82:1. The mean age of participants was 6.29 ± 1.35 (5-9) years.

**Renal involvement:** At the time of diagnosis, the renal involvement was discovered in only one patient as microscopic hematuria. During the six-month follow-up, 14 out of 48 patients including the first case (29.16%) developed to renal involvement. Ten out of 14 affected patients were boys with a boy to girl ratio of 2.5:1 (table 1). The association between sex and renal involvement was not significant (P=0.17). The mean age of patients with renal involvement was higher than patients without renal involvement (7.71±1.2 vs 5.7±0.9; P=0.001). 

**Table 1:** The relationship between sex, age, and serum aPL antibodies with renal involvement of IgA vasculitis patients

**Variable**		**Number with renal involvement (%)**	**Number without renal involvement (%)**	**Total**	**P-value**
**sex**	**Boy**	10 (71.4%)	17 (50%)	27 (56.2%)	0.174
	**girl**	4 (28.6%)	17 (50%)	21 (43.8%)	
**aPL**	**Positive** ^1^	9 (64.3%)	6 (17.6%)	15 (31.2%)	0.002
	**negative**	5 (35.7%)	28 (82.4%)	33 (68.8%)	
		**mean± SD**	**mean± SD**	**mean± SD**	**P.V**
**Age (years)**		7.71±1.2	5.7±0.9	6.29±1.35	0.001

^1^: More than 20 unit in anti-cardiolipin and beta-2 glycoprotein antibodies or lupus anticoagulant positivity

All renal complications appeared within six months during the course of IgA vasculitis and included hematuria and proteinuria. All 14 patients with renal involvement showed hematuria, while just two cases had gross hematuria. Five out of them presented with additional proteinuria, while only one patient developed to nephrotic-range syndrome.

**Antiphospholipid antibodies in IgA vasculitis children:** At the time of admission, the aPL antibodies were present in 15 (31.2%) patients, of which 9 (18.8%) patients were Lupus anticoagulant (LAC) positive, 4 (8.3%) patients had aCL-IgM, 3 (6.3%) patients had anti-beta-2 glycoprotein IgM, and 2 patients had aCL-IgG. The results of the second aPL tests that were performed again 12 weeks apart were complies with the previous investigation and were positive in the 15 positive patients and negative in the 33 negative patients. Antiphospholipid antibodies were positive in nine patients with IgA vasculitis who showed renal involvement (9 out of 14, 64.28%) during the follow-up period, compared with only six patients with IgA vasculitis without renal involvement (6 out of 34, 17.64%). Hence, the positive percentages of lupus anticoagulant, anti-cardiolipin antibodies, and anti-β2 glycoprotein antibodies (antiphospholipid antibodies) were much higher in serum of children with renal involvement (p<0.01), and there was a moderate correlation between positive aPL antibodies and renal involvement, with a kappa index of 0.457 (table 1).

Based on these findings, nine patients with renal involvement had aPL antibodies (64.3%) and six patients without renal involvement had aPL antibodies (17.6%). The presence of aPL antibodies was statistically associated with renal involvement, (P=0.002). Among aPL positive patients, 12 out of 15 (80%) were males, and 18 out of 33 (54.5%) patients who were aPL negative were girl (table 2). The association between sex and aPL positivity was statistically significant (P=0.025). Similarly, the association between age and aPL positivity was statistically significant (P=0.046).

**Table 2:** The relationship of sex and age with aPL positivity

**Variable**		**aPL (+)** ^1^ ** numbers (%)**	**aPL (-) numbers (%)**	**Total**	**P-value**
**sex**	**Boy**	12 (80%)	15 (45.5%)	27 (56.2%)	0.025
	**girl**	3 (20%)	18 (54.5%)	21 (43.8%)	
**Variable**		**mean± SD**	**mean± SD**	**mean± SD**	**P.V**
**Age (years)**		6.86±1.45	6.03±1.23	6.29±1.35	0.046

The relationship between variables in two groups have been shown in table 3, where based on multivariable analyzes, the sex variable was not significant between the groups. Both crude and adjusted odds ratios for estimating risk and controlling for confounding bias were evaluated in this study. According to modified logistic regression test that was modified based on the variables of sex, age and aPL positivity, the OR between groups with and without renal involvement was again insignificant in term of sex, but was significant in term of age and aPL positivity (table 3). 

**Table 3:** Determining the relationship between variables in case and control groups

	**Crude**			**Adjusted**		
**Variable**	**P.V**	**OR**	**CI (95%)**	P.V*	OR*	**CI (95%)** ^1^
**Sex**	0.18	2.5	0.64-9.55	0.13	0.08	0.003-2.13
**Age (year)**	0.001	0.22	0.1-0.5	0.004	0.12	0.02-0.51
**aPL positivity** ^2^	0.003	8.4	2.06-34.21	0.01	22.52	1.78-284.54

^1:^ based on logistic regression that was adjusted by age, sex, and aPL positivity.

^2^: More than 20 unit in anti-cardiolipin and beta-2 glycoprotein antibodies or lupus anticoagulant positivity

## Discussion

During the six-month follow-up, 14 out of 48 patients including one case who involved at presentation (29.16%) with a boy to girl ratio of 2.5:1, developed to renal involvement. The mean age of patients with renal involvement was higher than patients without renal involvement (7.71±1.2 vs 5.7±0.9; P=0.001).

Antiphospholipid antibodies were positive in nine patients with IgA vasculitis who showed renal involvement (9 out of 14, 64.28%) during the follow-up period, compared with only six patients with IgA vasculitis without renal involvement (6 out of 34, 17.64%). The positive percentages of antiphospholipid antibodies were much higher in serum of children with renal involvement (P=0.002), so that there was a moderate correlation between positive aPL antibodies and renal involvement, with a Kappa index of 0.457.

Although, the relationship between both sex and age with aPL positivity was statistically significant, performing a modified logistic regression test, the odds ratio was significant between the groups with and without renal involvement only in term of age and aPL positivity.

However, despite the self-limiting entity of IgA vasculitis in the majority of cases, the long-term prognosis is primarily depends on the severity of renal involvement, which may manifest as persistent hematuria or proteinuria, nephrotic or nephritic syndrome, or even End Stage Renal Disease (ESRD) (15). There are some interventions, like leukocytapheresis or medications such as cyproheptadine, which can prevent or at least decrease the occurrence of renal involvement in patients with IgA vasculitis. To be effective and workable, these interventions need a strong initial suspicion of renal involvement in IgA vasculitis (6). 

Early diagnosis is partly relying on knowing the risk factors for renal involvement. Wang et al. in a retrospective review on Chinese children, reported several risk factors for renal involvement in IgA vasculitis patients, including onset at an older age, colder season, longer interval between symptom onset of IgA vasculitis and its diagnosis, rural residency, skin lesion recurrence, angioedema and CNS involvement (1). Several of these reported risk factors have just a statistical relationship, nor a cause-effect relationship.

Chan et al., in a meta-analysis collected some factors associated with renal involvement in IgA vasculitis, such as older ages at onset, male gender, digestive tract symptoms and their severity, persistence (for more than one month) or recurrence of purpura, WBC >15×10^9^/L, platelet count >500×10^9^/L, upper respiratory tract infection, especially streptococcal infection with elevated ASO, and decreased C3 level (6).

De Almeida et al., in a retrospective study, analyzed the initial prognostic factors for renal involvement in IgA vasculitis and could find the severe abdominal pain among several risk factors as the only independent variable associated with nephritis, not other forms of renal involvement (16). Similarly, the age at diagnosis and abdominal pain were independent risk factors for renal involvement in Carucci et al.’s study (15).

Kim et al., in a medical records reviewing of children diagnosed as having HSP between 2005 and 2020, assessed risk factors associated with renal involvement in 186 patients. They found that blood neutrophil/lymphocyte ratio (P=0.002) and platelet/lymphocyte ratio (P=0.002) were significantly higher that patients without renal involvement. No statistically significant differences were found in the hemoglobin concentration, platelet count, presence of arthralgia, and gastrointestinal involvement between two groups. However, in logistic regression analysis, the female sex (OR=3.213) and neutrophil/lymphocyte ratio (OR=1.329) observed as the risk factors for renal involvement (4). 

Predictive value of laboratory indexes on renal involvement in 146 children with HSP in a Chinese study was performed. In this study, age, platelet distribution width (PDW), CD3^+^_, _and fibrinogen were positively correlated with renal involvement, while the values of C-reactive protein (CRP), Immunoglobin G, and neutrophil/lymphocyte ratio were negatively correlated with renal involvement (17). 

 There have been controversies on the treatments especially corticosteroids in preventing the course of renal involvement in more recent studies. Nevertheless, Chartapisak et al., in a review, analyzed the available articles and found that corticosteroids do not prevent or alter the course of renal involvement in IgA vasculitis (3). In 2014, Davin et al. reported that prompt institution of aggressive immunosuppressive treatment could blunt the risk of renal disease progression (18). This finding, although not strongly proven yet, has an important implication that if the patient with IgA vasculitis is treated early and correctly, the risk of renal involvement will be decreased, underscoring the importance of identifying the risk factors.

Among the studies reporting these risk factors, we could not find any humoral marker associated with an increased risk of renal involvement. There are several reports of association of antiphospholipid antibodies with IgA vasculitis disease and different manifestations of this disease. Basaran Kaya et al., and Yang et al., reported that IgA aPL antibodies to be associated with IgA vasculitis disease activity. They proposed that aPL antibodies may play a role in IgA vasculitis onset (9,19). 

Kawakami et al., reported the association between high titer serum aPL and cutaneous leukocytoclastic angiitis in IgA vasculitis patients (20). Sokol et al., reported the association of serum and CSF aPL titers with neurologic involvement in IgA vasculitis patients (21). Whether IgA vasculitis has a coincidental association with aPL antibodies is still poorly understood; however, it seems to be more than a coincidence. IgA vasculitis-related antibodies cause vasculitis, which exposes the endothelial phospholipids, otherwise non-exposed, allowing aPL antibodies to interact with them (22). 

Our results show a moderate correlation between serum aPL antibodies and renal involvement. This correlation can not only show the positive serum aPL antibodies as a risk factor for renal involvement in IgA vasculitis patients but can also show a cause-effect relationship. In other words, this correlation may propose the aPL antibodies as a causative factor for renal involvement in IgA vasculitis. 

This may have important diagnostic and treatment implications. Literature shows an earlier diagnosis of renal involvement leads to more rapid treatment establishment, which clearly improves the prognosis. If positive aPL antibodies can predict renal involvement in IgA vasculitis, it can help the earliest treatment, hence improving the prognosis more. 

If we consider positive serum aPL antibody as a predictable factor of renal involvement in IgA vasculitis, our study shows a sensitivity of 64.3%, a specificity of 82.4%, a Positive Predictive Value (PPV) of 60.0% and a Negative Predictive Value (NPV) of 84.8%, showing that a positive serum aPL antibody can be used to positively predict the renal involvement, while a negative result is not strong enough to rule out future renal involvement.

This study can only open a new vision into the correlation of aPL antibodies and renal involvement in IgA vasculitis patients. The main limitation of this study is the limited number of sample size and lack of renal biopsy as a diagnostic and follow-up tool for renal involvement since renal biopsy was not part of our management protocol for renal complications in IgA vasculitis. Hence, we need multicenter, more extensive cohort studies to reach a better and more accurate conclusion on the relationship between serum aPL antibodies and renal involvement in IgA vasculitis patients. 

In this study, we found that aPL antibodies were statistically associated with renal involvement. Positive aPL antibodies in a patient with IgA vasculitis can be considered as an alarm for probable renal involvement during the course of IgA vasculitis, necessitating closer screening and follow-up for renal involvement in these patients, to diagnose and treat the nephritis earlier. 
